# Web-Based Patient-Reported Outcomes for ENT Patients—Evaluation of the Status Quo, Patients’ View, and Future Perspectives

**DOI:** 10.3390/ijerph191811773

**Published:** 2022-09-18

**Authors:** Theresa Wald, Veit Zebralla, Maren Boege, Viktor Kunz, Thomas Neumuth, Andreas Dietz, Gunnar Wichmann, Susanne Wiegand

**Affiliations:** 1 Department of Otorhinolaryngology, Head and Neck Surgery, University of Leipzig Medical Centre, 04103 Leipzig, Germany; 2 Innovation Center Computer Assisted Surgery, University of Leipzig, 04103 Leipzig, Germany

**Keywords:** eHealth, patient-reported outcome, tumor aftercare, web-based, head and neck cancer, new media

## Abstract

Background: Patient-reported outcomes (PRO) assess disease burden and indicate unmet needs. Home-based electronic PRO measures (ePROMs) can support tumor aftercare (TAC). Creating an ePROM is the next step after implementing the software “OncoFunction” to assess PROs during TAC of head- and neck-cancer patients (HNC). Therefore, internet use and perception on ePROMs of ENT and TAC patients were evaluated. Methods: From May–July 2020, ENT patients at a high-volume outpatient department aged >18 without need for emergency treatment were invited to complete a questionnaire concerning internet use and access, hardware, and opinion on the chances, requirements, and designs of ePROMs. Results: 415 questionnaires were evaluated; 46.3% of the respondents visited the common consultation hour (CCH) and 44.3% TAC; 71.9% were internet users, being younger than non-internet users; and 36.4% of TAC patients were non-internet users and 16.3% of them were without a web-enabled device. Significant differences existed in age and assessment of future perspectives between internet-/non-internet users and TAC/CCH patients, respectively. Regarding the design of ePROMs, patients preferred quarterly and short surveys. Data safety and feedback were important. Conclusions: ePROMs are not suitable for everyone because of missing internet access and experience. A tailored approach to implement ePROMs in TAC is needed.

## 1. Introduction

In most cancer centers, tumor patients are assigned to tumor aftercare (TAC) after completion of tumor therapy according to the NCCN guidelines [1,2,3], aiming at achieving the longest survival and best quality of life (QoL). TAC is essential for detecting potentially recurrent diseases and monitoring early and late therapy-associated toxic side-effects at the earliest time possible to interfere by applying the indicated therapies. In patients with head and neck cancer (HNC), commonly reported side-effects after therapy are swallowing disorders, voice impairments, dry mouth (xerostomia), and pain, as well as psychological problems like depression, fatigue, and anxiety [4,5,6].

Clinicians may have a different view of the patient’s impairments and underestimate or miss symptoms by only evaluating clinical, biochemical, or radiological abnormalities [7,8,9,10]. Nonspecific symptoms or symptoms that are not directly related to the physician’s specialty, might not be detected and discussed if the patient does not report his complaints himself and the physician focuses on his field of expertise during the time-limited TAC visit, missing essential information about functional aspects [11,12].

With a patient-reported outcome measure (PROM), patients can report their perceptions of their individual physical, functional, emotional, social, health status, and QoL in a structured manner. Patient-reported outcomes (PROs) are increasingly used in clinical trials as an outcome measure [13]. The US Food and Drug Administration (FDA) defines a PRO as “any report of the status of a patient’s health condition that comes directly from the patient, without interpretation of the patient’s response by a clinician” [14] and provides criteria characterizing an appropriate PROM. Summed up, these criteria contain a reasonable PROM design (length, literal speech, and number of items), validity (Does the PROM measure the concept of interest?), reliability (Are the results of the PROM consistent and reproducible?), ability to detect a change in symptom/disease control over time (traceability), and usability (Are the items understandable? Are the respondents able to answer them (physically, mentally, as illiterates, as non-native speakers, etc.)?).

In a systematic review [15], the authors found evidence that routine collection of PROMs leads to an improvement in patient–provider communication and patients’ satisfaction. They also describe improved monitoring of treatment response, increased symptom control, and detection of unmet needs. PROs might contribute to patient-centered medicine and shared decision-making. Referring to Basch et al. [16], regular completion of PROMs with an alert in case of symptom abnormalities can improve overall survival in patients with metastatic solid tumors. PROMs can provide a comprehensive approach to unmet needs and show the need for action [11].

At our department of otorhinolaryngology (ENT; ear, nose and throat), a software tool named “OncoFunction” was implemented in 2013 to incorporate PROs into each visit of TAC [17]. Using “OncoFunction”, PROMs were collected addressing important issues of patients after treatment for HNC such as pain, swallowing, voice and breathing, psychological and psychosocial terms, and other problems [4,5,6,18,19,20]. Courses of adverse effects (especially late toxicity related to particular treatment regimens) can be monitored over time [21].

To take one step further, web-based online PROMs (electronic PROMs, ePROMs) are on the rise. Home-based questionnaires give insight into patients’ symptom burden in their familiar surroundings, monitor their quality of life at home, detect changes, and allow the execution of decentralized trials. Not only in pandemic times with tumor patients being at risk of severe courses of infectious diseases like COVID-19 and thus aiming for a reduction of patient-physician-contacts to a minimum, but ePROMs could also help to identify patients’ problems and understand the perspective of the patient. To be clear: ePROMs are designed to support and closely monitor the patient’s disease course and not to replace regular clinical visits.

The aim of this study, therefore, was to evaluate the risks and chances of implementing new media into patient care. To design an easy-to-use, easily accessible, and highly accepted ePROM, it should be analyzed how ENT patients with and without HNC access the internet and how they use it. Moreover, the patient’s perception of potential advantages and requirements of home-based online tools is of interest and advices should be evaluated. Differences between HNC patients visiting TAC and patients with other ENT diseases visiting a common consultation hour (CCH), as well as internet and non-internet users, are especially relevant and, hence, deserve particular attention.

Thus, we were interested in the questions: (i) Do our patients and especially HNC patients have access to the internet? Do they use the internet for answering health-related questions? (ii) Are there differences between cancer and non-cancer patients? (iii) How far are patients willing to share personal and health data via the internet? What is their attitude toward eHealth? (iv) How should an online tool to assess patient-reported outcomes (ePROM) be designed?

## 2. Materials and Methods

Within a quality-assurance measure from May–July 2020, patients at the ENT outpatient clinic of a university hospital were asked to complete a questionnaire consisting of 35 questions concerning internet use (quantitatively and qualitatively, potential time spent, and sources used for information on health/disease and treatment), digital equipment, willingness to share personal data, and requirements for potential ePROMs. The questionnaire is available in the Supplementary Material section (Supplement S1).

Paper-based questionnaires were administered to every patient after registration at the ENT outpatient clinic and were collected by the nurses before or after the doctor’s visit. Exclusion criteria were age <18 years, a need for urgent emergency treatment, illiterate patients, and a patient’s refusal. To receive comparable data from tumor and non-tumor patients, we only invited patients to answer the questionnaire on days when both, the TAC and the CCH, took place in our ENT department.

The Ethics Committee of the University Hospital Leipzig approved analyzation and publication of the collected data (file number 447/20-ek).

Information on demographics, internet use, and potential data-sharing regarding ePROM was recorded. We performed statistical analysis via SPSS version 27 (IBM Corp. Released 2020. IBM SPSS Statistics for Windows, Version 27.0. Armonk, NY, USA) using Pearson’s χ^2^- and Fisher’s Exact- and t-test to evaluate the differences among internet users and non-internet users, as well as the differences between non-tumor patients of the CCH and tumor patients of the TAC. *p* values < 0.05 were considered statistically significant. A Bonferroni correction was applied in the case of multiple comparisons. In cases of missing values, we used pairwise deletion to only consider available cases.

## 3. Results

419 questionnaires were evaluated. Four questionnaires were excluded from the analysis as they did not fulfill the inclusion criteria because of age and might have been handed out by accident to a parent. In total, 192/415 (46.3%) of patients visited a doctor for common ENT issues during the CCH, 184/415 HNC patients (44.3%) visited the department for TAC, and 39/415 (9.4%) did not provide their reason for consultation. In the following, the readers need to be aware that using pairwise deletion to consider missing values results in different total numbers.

In total, 264/400 (66%) of the patients were male and 136/400 (34%) were female. The chronologic ages ranged from 20 to 90 years (mean 58.2 years; standard deviation (SD) 16 years). General information about patients is shown in Table 1. Table 2 introduces the status quo of internet access and use among CCH and TAC patients, as well as internet and non-internet users. Table 3 indicates the patients’ perception of ePROMs and their design, evaluated in the subgroups of CCH and TAC patients, as well as internet and non-internet users.

According to the visits of TAC in the mentioned time interval, the collected questionnaires cover at least 76% of TAC patients. The response rate of CCH patients settles above.

### 3.1. Internet Access and Use of the Whole Cohort of ENT Patients

Most patients used the internet (297/413, 71.9%). Smartphones or computers were the main sources for gaining access to the internet. Patients often owned more than one web-enabled device (196/414, 47.3%), and 54/414 patients (13.0%) did not own any internet-enabled device. Ownership of more than one device was associated with a higher education level, e.g., university degree and younger age (both *p* < 0.001). Non-internet users were significantly older than those using the internet (mean age 67.8 (SD 11.6) vs. 54.4 (SD 16.0) years; *p* < 0.001). The most common reasons for spending time on the internet were searching for information, communication via email, and online shopping; 59.6% of the patients used the internet for less than 2 h a day and 74% for less than 4 h a day for private concerns (*n* = 366); 185/352 (52.6%) patients did not need the internet for work at all, whereas 98/352 (27.8%) spent less than 2 h per day on the internet for work-related tasks.

Concerning health issues, many patients got advice and information exclusively from their doctors and did not spend time on the internet for health-related research (259/373, 69.4%). Most patients had never visited a medical website (237/349, 67.9%).

### 3.2. Internet Access and Use by HNC Patients vs. Other ENT Patients

**Figure 1:**
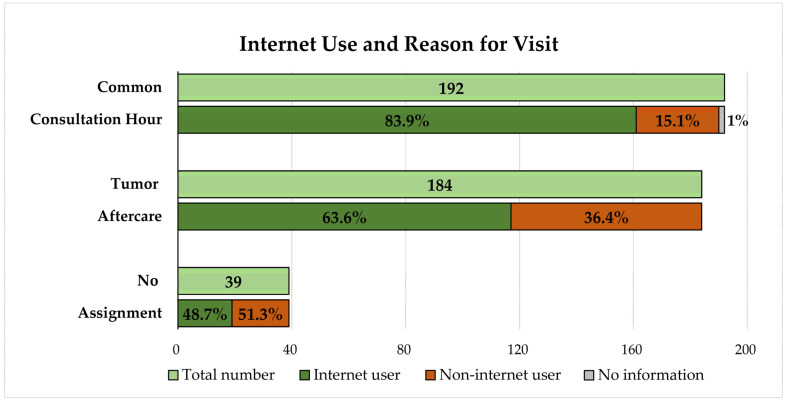
Visualization of the distribution of study participants regarding reason for visit and internet use. Internet users are colored in dark green, non-internet users in orange. Missing information regarding internet use is colored in grey. The bar in light green visualizes the number of patients in each patient group and equals 100% of the group. Thirty-nine patients did not provide their reason for visiting.

There were significant differences between the groups of patients admitted to TAC and CCH for other ENT issues (Table 2). Compared to CCH patients, more TAC patients were male (73.9% vs. 55.2%) and of higher age (mean 64.3 (SD 10.6) vs. 51.4 (SD 17.65) years); 30/184 (16.3%) TAC patients vs. 13/192 (6.8%) CCH patients did not own a web-enabled device. After applying the Bonferroni correction, the level of education did not remain significant. 67/184 (36.4%) TAC patients and 29/192 (15.1%) CCH patients negated using the internet (all *p* < 0.001). In Figure 1, the distribution between either CCH or TAC and internet use is visualized. 

Searching for information, emailing, and shopping were the most common internet activities in both groups. Both groups differed significantly in the amount of time spent on the internet, but they do not spend a significantly different amount of time on seeking information concerning health-related issues.

### 3.3. Chances and Future Imagination of ePROMs from the View of the Whole Cohort of ENT Patients

Several items of the questionnaire evaluated the patient’s attitude toward ePROMs, including the design of recurrent questionnaires and engagement in using electronic applications reporting patient data (Table 3). In total, 206/406 (50.7%) ENT patients would accept a device-based application (APP) as a communication tool. If suffering from a chronic disease, 52.8% of ENT patients prefer an interval of disease monitoring via questionnaire every three months or less. In the opinion of 71% of the respondents, the questionnaire should take less than 10 min, and 84.2% of the patients think that an ePROM should consist of fewer than 20 questions. In all, 211/364 (58.0%) are confident to collect health data (e.g., heart rate and blood pressure), possibly by APP support, and 178/362 (49.2%) assume an improvement in patient care by using an APP in addition to regular consultations with physicians. Replacing consultation hours with online consultations is refused by 238/381 (62.5%) patients. Data safety, keeping their own data under control, and receiving a response to the information sent via APP are desired and/or seen as requirements for agreeing to transfer personal data via APP. However, 162/339 (47.8%) would like to make appointments via email, especially those of younger age (*p* < 0.001) or with a university degree (*p* = 0.004).

### 3.4. Patients’ View on Chances and Future Prospects of ePROM Use

The patient’s view on reporting personal data via electronic application(s) differs when dividing ENT patients into subgroups of tumor patients and non-tumor patients. The majority of TAC patients cannot imagine communicating via APP with their hospital (103/181, 56.9%), whereas most CCH patients can (116/188, 61.7%) and only 38.3% cannot envisage digital communication (72/188; *p* = 0.001). Thus, more TAC patients than CCH patients do not want to replace physical doctor’s appointments with an online consultation (127/175, 72.6%, vs. 87/170, 51.2%; *p* < 0.001). Moreover, the benefits of using an APP, the capability of collecting their own health data, and the willingness to transfer data via APP to a hospital are judged differently by TAC and CCH patients (all *p* < 0.004). Interestingly, CCH patients preferred shorter intervals between surveys in cases of suffering from a chronic disease compared to TAC patients (*p* < 0.001). There were no differences in the patients’ views on the design of the questionnaire (*p* > 0.15) (Table 3).

## 4. Discussion

In this cross-sectional, mono-centric study, internet access and activities of ENT patients visiting CCH and TAC were assessed and their perceptions of the design and future perspectives of ePROMs were evaluated. There were significant differences in chronologic age, and assessment of future perspectives according to internet use (yes vs. no) and the reason for consultation (TAC vs. CCH). As HNC affects more men, the frequency of male participants consulting their physician for TAC after the cure of their HNC was higher and correlated with the higher number of questionnaires completed by men. The majority of ENT patients in this study used the internet or owned at least one web-enabled device. Internet users were significantly younger than non-internet users, just as CCH patients compared to TAC patients. The internet was mostly used for seeking information not related to health information, which, as such, was mostly provided by the treating physician. About 50% of the ENT patients have a positive attitude toward using an APP for PRO. Data safety and receiving feedback are very important for the patients when completing a PROM. TAC patients seem to be more dismissive and skeptical in their perceptions concerning the use and benefit of ePROMs.

PROMs in general give insight into the patient’s problems in daily life caused by functional and physical impairment. Provided unlimited and barrier-free access to devices and their connectivity, regular use of home-based ePROMs, reported in the everyday surrounding of the patient, might be best to capture problematic areas. The results of this study are helpful when designing an ePROM and implementing it in the clinical routine. As not every patient is able to access the internet and answer appropriately, or to handle the recording of relevant issues, caution is advised before trying to implement an ePROM. Nevertheless, several studies indicate the benefit of PROMs, showing that creating an ePROM is worth the effort. Besides improved symptom control and health-related quality of life [22], Basch et al. reported a significantly increased overall survival and prolonged treatment with chemotherapy of patients with metastatic breast, genitourinary, gynecologic, or lung cancers [16]. Their patients were assigned to either a computer-experienced or computer-inexperienced group and within these groups either to usual care or the intervention arm with self-reported PROs. Randomized to the intervention arm, computer-experienced patients had to complete weekly home-based ePROMs, in addition to the PROMs at each clinical visit, while the inexperienced completed PROMs only at the clinical visit. Nurses intervened when alerted via email in cases of severe symptom worsening. Health-related quality of life was less impaired in the intervention arm but without statistical significance in the subgroup of computer-inexperienced patients with balanced baseline characteristics between both subgroups. However, selection bias cannot be excluded whenever patients are stratified into experienced vs. inexperienced because of substantial confounding.

Remarkable findings were also reported in a study by Denis et al. [23,24]. As these authors recognized that some patients who experience an increase in symptom burden did not contact a doctor but waited until their regular TAC appointments, e.g., in 3 months, to discuss their impairment, they chose weekly intervals to monitor symptom burden in detail via ePROMs. In a randomized controlled trial with 133 patients with metastatic lung cancer, the intervention group completed ePROMs weekly. By alerting the oncologic staff whenever the symptom burden increased, they triggered earlier utilization of appropriate treatment intervention and achieved a significantly better overall survival (OS) than the control group (OS 22.5 months vs. 14.9 months, *p* = 0.03). In the intervention group, less-regular CT scans were made because of the results of earlier studies when an increase in symptom burden has indicated a relapse better than the regular CT scans (every 3–6 months in the control arm and every 6 months in the intervention arm) [25,26]. Additionally, a cost benefit was described when using web-based PRO surveillance in patients with lung cancer [27].

Until now, it is unclear if PROs have a positive effect on the overall survival of HNC patients and further studies are needed. Nevertheless, it is essential to identify patients at risk of having no benefits from implementing ePROMs as they are, for instance, visually impaired, functionally illiterate, or otherwise handicapped and, hence, not able to appropriately use them.

The majority of our patients had access to the internet (333/376, 88.7%) and used it regularly (297/413, 71.9%). Of the non-internet users, 42.2% (49/116) did not own a web-enabled device and, thus, definitely did not have any access to the internet. Non-internet users being older than internet users on average in this study is in line with the findings of Wintner et al., who report a strong skepticism of older patients toward eHealth [28]. They assumed that older people are less familiar with online activities and, because of missing online skills, are skeptical and refuse eHealth. Exploring the needs of supportive care, Jansen et al. [29] also reported an association of lower age with a positive attitude toward self-management and eHealth.

By contrast, there is a growing (and aging) population using new media and even among the oldest there are many being well-educated in using the internet. Our data suggest that especially younger (cancer) patients are more familiar with online activities and might benefit from the implementation of an online tool, as they are more active and/or employed. To them, data transfer is conceivable if data safety and receiving feedback to their data are guaranteed.

Currently, one-third of the presented HNC patients do not use the internet, and thus collecting health data via app or using ePROMs will be difficult. Their motivation appears to be limited as they do not see any advantage of using an app in addition to regular visits; 56% of TAC patients cannot imagine communicating with their physician via APP. These circumstances call for a tailored approach concerning the implementation of new media in TAC or health care in general, with training and assistance offered to introduce and/or support the completion of the ePROM meeting the individual needs of particular patient groups. Support is especially needed when patients are in poor general condition, have speech problems, or are simply not familiar with the language, illiterate, visually impaired, or physically not able to handle an internet-enabled device. Technical problems demand special support.

A Dutch study on HNC patients after total laryngectomy reported similar findings concerning internet use [30]: 68% of the patients reported regular use of the internet, especially those of younger age and with better education, and those with shorter time after a laryngectomy. According to these authors, eHealth, besides recording PROs, might provide tools to inform about the disease and serve patients in the difficult phase directly after laryngectomy. A weakness of their study is the systematic selection of computer-friendly patients, omitting all others. According to the study design, questionnaires were sent to 944 patients with a history of total laryngectomy; 288 questionnaires were sent back without tracking or making inquiries of the non-respondents, leading to a systematic selection of compliant and motivated patients and a serious selection bias allowing only conclusions for a “best-case scenario”. Our study covers a raw sample of ENT patients in general and TAC patients. There is no selection of computer-experienced and new-media-friendly or compliant patients in our study because every patient entering the outpatient department was invited to participate via face-to-face contact with the study nurse to answer the questionnaire during the waiting time before entering the doctor’s room.

Our cohort differs from the standard German population according to internet use. In an annual survey conducted by the most important German public-service television broadcasters ARD/ZDF via telephone interview [31], 94% of the Germans affirm using the internet. Thus, internet use is less common in our cohort of ENT patients (71.9%). In the group aged >70 years, 77% of the general German population are internet users. Of the elderly (≥70 years) in our cohort, internet users were less frequent (53.9%; 48/89), and according to the reason of visit, 58.8% were in CCH (20/34) and only 50.9% (28/55) in TAC. Comparing these frequencies with the general German population indicates ENT patients in general, and especially TAC patients, are a vulnerable group differing from the general population regarding internet use and willingness to use ePROMs. Thus, specialized and custom-tailored approaches are needed and ePROMs cannot be used to replace regular visits.

From our patients’ point of view, a good ePROM may contain up to 20 questions that can be answered in less than 10 min every 3 months. Patients are willing to transfer their data if (1) data safety is guaranteed (CCH and TAC patients), (2) they receive feedback or even an answer to their query (CCH and TAC patients), and (3) they can access their prior data and incrementally alter it, or if (4) they have a personal advantage (CCH patients) or benefit by reducing the frequency of their doctor appointments (TAC patients). Simultaneously, 72.6% (127/175) of the TAC patients cannot imagine replacing the physical consultations with the treating physician, including medical examinations, with online consultations.

We decided to use pairwise deletion in statistical analysis to cover a raw sample of ENT patients. To prevent missing values, a notification for missing answers could be inserted into an online tool; in general, questionnaires should be kept short and easy to understand. Comparing paper-based and electronic questionnaires, Kongsved et al. [32] found a higher response rate to paper-based questionnaires sent via postal service to the patients compared to those who answered an online questionnaire. To achieve a sufficient response rate of the online questionnaires, the authors had to send at least one reminder to the patients. On the other hand, online questionnaires were complete in 97.8% of the cases without missing values, compared to 63.4% of the paper-based questionnaires. Additionally, web-based PROMs were more cost-effective than paper-based questionnaires. Because of the higher completion rate using electronic PRO, the regular PRO-assessment during TAC in our clinic using the software “OncoFunction” is tablet-based (and offers the advantage of easy recording and lossless storage of PRO without any form of interpretation and confirmation bias).

For a successful implementation and acceptance of ePROMs by patients, feedback given directly, or at least within the near time, by the physician is desired to keep the patients’ willingness to further enter their data. Therefore, editing and replying to ePROMs must be integrated into the clinical routine to not be missed when the patient is not physically present. Designing an interactive response system that immediately provides recommendations, such as, for instance, to proceed by arranging an appointment with the treating ENT specialist, might be helpful to maintain adherence to TAC programs offering partial replacement of regular visits by ePROM assessments and, moreover, to transfer electronic alerts into actions when needed. In particular, disabled and immobile patients living in rural regions may benefit from recording their data and interactive response systems before seeking direct interaction and examination by their physician without prior notice. Trained (oncology) nurses could sift the alerts and assign the patient to a matching path as realized and described by Basch et al. [22]. The software “OncoFunction” already provides a traffic light alert system when PROs are worse compared to their last visit and might be suitable for ePROM in an adapted form alerting the involved ENT specialists.

Currently, a relevant number of HNC patients are not familiar with internet and web-based solutions. These patients need special care in daily clinical practice. Arranging regular telephone calls with patients who are not familiar with the internet and web-based solutions would be necessary to get PROM data instead of ePROM data. This solution might be more expensive than web-based solutions but may minimize the risk of losing those patients who require the TAC the most, for instance (functional) illiterate patients, from aftercare.

There are some limitations to our study. First, the used questionnaire is not validated. Within a quality measure, we were interested in the patients’ opinion to gain experience and identify potential pitfalls concerning implementing new media into patient care. Because the questionnaire will not be used for other concerns outside of the quality measure, a detailed validation is time- and resource-consuming with little to no benefit. The included questions were short and were expressed in simple language. In fact, by using written questionnaires, a potential bias created by excluding illiterate patients may remain. This may result in an underestimation of proponents of ePROMs and, thus, underline our call for a tailored approach when implementing ePROMs or new media in TAC.

Many questionnaires were not filled in completely. Questions especially in the middle or at the end of the questionnaire have been skipped, potentially because patients were overloaded with information and, thus, lost concentration and interest in answering the questions. This goes in line with the preference of many patients for questionnaires requiring little time to complete. Furthermore, sometimes questions printed on the back side of the paper may have been overlooked.

Because the presented survey was part of a quality measure, we went without secondary information about other patients’ characteristics such as level of income, profession, relationship status, smoking, and alcohol consumption, or detailed information on medical conditions. The questionnaire was designed only to obtain the essential minimum of personal data to assess the patients’ views on ePROMs and the feasibility of their implementation. Nonetheless, we gained clear information on the subject of interest as described above, even if only core data were used. Obviously, for developing a validated tool to assess ePROs, further detailed information is needed but was beyond the investigators’ point of interest at the time the study was conducted.

## 5. Conclusions

PROMs promise improved patient-centered health care and symptom monitoring. The majority of patients has access to the internet and is familiar with its use. Many patients are open-minded to completing ePROMs regularly in cases of chronic disease. Nonetheless, a significant number of HNC patients have neither internet access nor are familiar with using online questionnaires and, thus, are skeptical concerning the use of new media in health care, including ePROMs. Therefore, a tailored approach is needed to implement ePROMs in TAC.

## Figures and Tables

**Table 1:** Characterization of the patient cohort stratified according to either reason for consultation (common consultation hour vs. tumor aftercare) or internet use (yes vs. no); 415 questionnaires were evaluated.

		Common Consultation Hour *		Tumor Aftercare *			Internet Users °		Non- Internet Users °		
		*n*	%	*n*	%	*p* Value	*n*	%	*n*	%	*p* Value
**Total *n***		192	100	184	100		297	100	116	100	
**Sex**	Male	106	55.2	136	73.9	**<0.001**	186	62.6	76	65.5	0.705
	Female	81	42.2	42	22.8		99	33.3	37	31.9	
	Missing values	5	2.6	6	3.3		12	4.0	3	2.6	
**Age** **categories** **[years]**	Mean	51.4		64.3		**<0.001**	54.4		67.8		**<0.001**
	Standard deviation	17.7		10.6			16.0		11.6		
	Minimum	20		29			20		40		
	Maximum	90		88			88		90		
	18 to 29	24	12.5	1	0.5	**<0.001**	26	8.8	0	0	**<0.001**
	30 to 39	30	15.6	2	1.1		35	11.8	0	0	
	40 to 49	39	20.3	10	5.4		45	15.2	7	6.0	
	50 to 59	34	17.7	47	25.5		65	21.9	21	18.1	
	60 to 69	29	15.1	66	35.9		68	22.9	37	31.9	
	70 to 79	24	12.5	37	20.1		41	13.8	26	22.4	
	>80	10	5.2	18	9.8		10	3.4	25	21.6	
	Missing values	2	1.0	3	1.6		7	2.4	0	0	
**Education**	Professional training	81	42.2	97	52.7	0.023 ^a^	129	43.4	71	61.2	**<0.001**
	Technical college	23	11.9	16	8.7		35	11.8	9	7.8	
	University of applied sciences	19	9.9	23	12.5		41	13.8	4	3.5	
	University degree	46	24.0	22	12.0		65	21.9	6	5.2	
	Other	10	5.2	14	7.6		12	4.0	12	10.3	
	None	9	4.7	5	2.7		12	4.0	4	3.5	
	Missing values	4	2.1	7	3.8		3	1.0	10	8.6	
**Internet use**	Yes	161	83.9	117	63.6	**<0.001**					
	No	29	15.1	67	36.4						
	Missing values	2	1.0	0	0						

Some respondents made no attribution to their * reason for consultation (*n* = 39) or their ° internet use (*n* = 2). Bold values denote statistical significance at the *p* < 0.05 level. ^a^ no significance after Bonferroni correction for multiple testing.

**Table 2:** Evaluation of internet access and internet use of ENT patients stratified according to reason for consultation (common consultation hour vs. tumor aftercare) and internet use (yes vs. no).

		Common Consultation Hour		Tumor Aftercare			Internet Users		Non-Internet Users		
		*n*	%	*n*	%	*p* Value	*n*	%	*n*	%	*p* Value
**Total *n***		192	100	184	100		297	100	116	100	
**Do you own a web-enabled device?**	Smartphone	59	30.7	63	34.2	**<0.001**	76	25.6	61	52.6	**<0.001**
	Tablet	2	1.0	2	1.1		4	1.4	0	0	
	PC	5	2.6	16	8.7		18	6.1	3	2.6	
	None	13	6.8	30	16.3		5	1.7	49	42.2	
	More than one	113	58.9	73	39.7		193	65.0	3	2.6	
	Missing values	0	0	0	0		1	0.3	0	0	
**Which operating** **system do you use?**	iOS	34	17.7	10	5.4	**0.001**	45	15.2	0	0	**<0.001**
	Android	80	41.7	72	39.1		152	51.2	11	9.5	
	Other	4	2.1	10	5.4		15	5.1	1	0.9	
	Don’t know	30	15.6	37	20.1		37	12.5	40	34.5	
	More than one	25	13.0	10	5.4		35	11.8	1	0.9	
	Missing values	19	9.9	45	24.5		13	4.4	63	54.3	
**Main reasons for** **being online?** **(Multiple answers possible.)**	Email	133	69.3	92	50.0	**<0.001**	235	79.1	5	4.3	**<0.001**
	Chatting	72	37.5	29	15.8		100	33.7	2	1.7	
	Online communities	54	28.1	20	10.9		77	25.9	2	1.7	
	Research	133	69.3	97	52.7		234	78.8	7	6.0	
	Shopping	98	51.0	59	32.1		166	55.9	2	1.7	
	Work tasks	83	43.2	28	15.2		113	38.0	3	2.6	
	Other	13	6.8	9	4.9		5	1.7	10	8.6	
	Not answered	34	17.7	60	32.6		19	6.4	91	78.4	
**Do you use the** **internet for research** **concerning health?**	Yes	65	33.9	41	22.3	**0.011**	112	37.7	2	1.7	**<0.001**
	No	108	56.3	125	67.9		171	57.6	87	75.0	
	Missing values	19	9.9	18	9.8		14	4.7	27	23.3	
**How many hours a day do you spend on the internet for private use on average?**	<30 min	25	13.0	29	15.8	**<0.001**	50	16.8	7	6.0	**<0.001**
	<1 h	31	16.2	36	19.6		67	22.6	4	3.5	
	1 to 2 h	48	25.0	37	20.1		90	30.3	0	0	
	2 to 3 h	24	12.5	10	5.4		35	11.8	0	0	
	3 to 4 h	12	6.3	3	1.6		17	5.7	1	0.9	
	4 to 5 h	5	2.6	0	0		6	2.0	0	0	
	5 to 6 h	6	3.1	1	0.5		7	2.4	0	0	
	>6 h	3	1.6	0	0		3	1.0	0	0	
	None	16	8.3	48	26.1		5	1.7	73	62.9	
	Missing values	22	11.5	20	10.9		17	5.7	31	26.7	
**How many hours a day do you spend on the internet for work-related tasks on** **average?**	<30 min	20	10.4	13	7.1	**<0.001**	33	11.1	2	1.7	**<0.001**
	<1 h	20	10.4	10	5.4		29	9.7	1	0.9	
	1 to 2 h	25	13.0	6	3.3		33	11.1	0	0	
	2 to 3 h	13	6.8	4	2.2		18	6.1	1	0.9	
	3 to 4 h	7	3.7	2	1.1		9	3.0	0	0	
	4 to 5 h	10	5.2	1	0.5		11	3.7	1	0.9	
	5 to 6 h	7	3.7	4	2.2		11	3.7	0	0	
	>6 h	13	6.8	5	2.7		17	5.7	1	0.9	
	None	58	30.2	109	59.3		113	38.1	72	62.1	
	Missing values	19	9.9	30	16.3		23	7.7	38	32.8	
**How do you get** **information concerning your health issue?** **(Multiple answers possible.)**	Doctors	155	80.7	151	82.1	0.025 ^a^	259	87.2	72	62.1	**<0.001**
	Friends	17	8.9	12	6.5		28	9.4	3	2.6	
	Magazines	6	3.1	14	7.6		17	5.7	4	3.5	
	Internet	78	40.6	54	29.4		135	45.5	2	1.7	
	TV	3	1.6	9	4.9		10	3.4	2	1.7	
	Other	5	2.6	6	3.3		8	2.7	3	2.6	
	Not answered	24	12.5	25	13.6		21	7.1	40	34.5	
**How much time do you spent on the internet for research concerning health** **issues?**	<1 h	76	39.6	59	32.1	0.710	116	39.1	26	22.4	**0.001**
	1 to 4 h	34	17.7	25	13.6		66	22.2	2	1.7	
	4 to 8 h	11	5.7	14	7.6		27	9.1	0	0	
	8 to 16 h	5	2.6	6	3.3		11	3.7	0	0	
	16 to 24 h	4	2.1	4	2.2		8	2.7	0	0	
	1 to 3 days	5	2.6	1	0.5		6	2.0	0	0	
	3 to 7 days	3	1.6	1	0.5		4	1.4	0	0	
	>7 days	10	5.2	9	4.9		19	6.4	0	0	
	Missing values	44	22.9	65	35.3		40	13.5	88	75.9	
**Please rate the information published on the internet.**	1 very good	3	1.6	2	1.1	0.222	5	1.7	2	1.7	**0.001**
	2	38	19.8	35	19.0		74	24.9	5	4.3	
	3	68	35.4	43	23.4		113	38.1	3	2.6	
	4	23	12.0	10	5.4		31	10.4	4	3.5	
	5	7	3.7	5	2.7		11	3.7	1	0.9	
	6 deficient	2	1.0	6	3.3		5	1.7	3	2.6	
	Missing values	51	26.6	83	45.1		58	19.5	98	84.5	
**Have you ever visited a medical website?**	Yes	68	35.4	40	21.7	**0.013**	110	37.0	2	1.7	**<0.001**
	No	105	54.7	112	60.9		170	57.2	66	56.9	
	Missing values	19	9.9	32	17.4		17	5.7	48	41.4	
**Did you search the web for information about your medical treatment?**	Yes	50	26.0	35	19.0	0.277	88	29.6	2	1.7	**<0.001**
	No	120	62.5	111	60.3		181	60.9	73	62.9	
	Missing values	22	11.5	38	20.7		28	9.4	41	35.3	
**Was the information on the web helpful?**	Yes	43	22.4	39	21.2	0.782	85	28.6	2	1.7	**<0.001**
	No	87	45.3	85	46.2		128	43.1	66	56.9	
	Missing values	62	32.3	60	32.6		84	28.3	48	41.4	
**Would you like to make appointments via email?**	Yes	103	53.7	50	27.2	**<0.001**	158	53.2	4	3.5	**<0.001**
	No	64	33.3	93	50.5		107	36.0	69	59.5	
	Missing values	25	13.0	41	22.3		32	10.8	43	37.1	

Bold values denote statistical significance at the *p* < 0.05 level. ^a^ no significance after Bonferroni correction for multiple testing.

**Table 3:** Opinion on and attributed chances linked to using digital media in health care for patient-reported outcomes (PRO) and designing ePROM (electronic PRO-measurement) of ENT patients stratified according to reason for consultation (common consultation hour vs. tumor aftercare) and internet use (yes vs. no).

		Common Consultation Hour		Tumor Aftercare			Internet Users		Non-Internet Users		
		*n*	%	*n*	%	*p* Value	*n*	%	*n*	%	*p* Value
**Total *n***		192	100	184	100		297	100	116	100	
**Can you imagine communicating via APP with your** **physician/hospital?**	Yes	116	60.4	78	42.4	**<0.001**	194	65.3	12	10.3	**<0.001**
	No	72	37.5	103	56.0		100	33.7	99	85.3	
	Missing values	4	2.1	3	1.6		3	1	5	4.3	
**How often would you like to answer a questionnaire as a patient with cancer or any other chronic disease?**	Once a week or more	11	5.7	3	1.6	**<0.001**	12	4	4	3.5	**0.002**
	At least twice a month	31	16.2	13	7.1		38	12.8	9	7.8	
	Once a month	47	24.5	34	18.5		77	25.9	11	9.5	
	Once every 2 months	8	4.2	15	8.2		17	5.7	8	6.9	
	Once every 3 months	29	15.1	78	42.4		74	24.9	40	34.5	
	Less frequently	37	19.3	35	19.0		52	17.5	31	26.7	
	Missing values	29	15.1	6	3.3		27	9.1	13	11.2	
**How much time would you spend on a recurrent online questionnaire?**	<2 min	14	7.3	12	6.5	0.148	21	7.1	7	6.0	0.336
	<5 min	52	27.1	29	15.8		75	25.3	12	10.3	
	<10 min	54	28.1	54	29.4		97	32.6	18	15.5	
	<15 min	24	12.5	29	15.8		45	15.2	11	9.5	
	<20 min	16	8.3	20	10.9		27	9.1	10	8.6	
	Missing values	32	16.7	40	21.7		32	10.8	58	50	
**How many questions would you agree to answer regularly?**	<10	77	40.1	60	32.6	0.204	107	36	44	37.9	0.051
	11–20	70	36.5	65	35.3		122	41.1	20	17.2	
	21–30	17	8.9	22	12.0		32	10.8	9	7.8	
	31–40	3	1.6	7	3.8		8	2.7	3	2.6	
	41–50	0	0	2	1.1		1	0.3	1	0.9	
	>50	0	0	1	0.5		1	0.3	0	0	
	Missing values	25	13	27	14.7		26	8.8	39	33.6	
**Do you think you could collect health data (e.g., state of health, heart rate, blood pressure, etc.), if necessary, supported by the APP?**	Yes	79	41.2	60	32.6	**0.004**	134	45.1	18	15.5	**<0.001**
	Rather yes	31	16.2	26	14.1		52	17.5	6	5.8	
	Undecided	21	10.9	15	8.2		32	10.8	5	4.3	
	Rather no	19	9.9	18	9.8		27	9.1	10	8.6	
	No	20	10.4	48	26.1		30	10.1	49	42.2	
	Missing values	22	11.5	17	9.2		22	7.4	28	24.1	
**Do you think that using an APP in** **addition to regular doctor visits would improve patient care?**	Yes	59	30.7	36	19.6	**<0.001**	86	29	16	13.8	**<0.001**
	Rather yes	43	22.4	28	15.2		72	24.2	4	3.5	
	Undecided	38	19.8	37	20.1		63	21.2	17	14.7	
	Rather no	12	6.3	16	8.7		23	7.7	7	6.0	
	No	18	9.4	48	26.1		30	10.1	44	37.9	
	Missing values	22	11.5	19	10.3		23	7.7	28	24.1	
**Would you use an APP to transfer your health data to your hospital?**	Yes	66	34.4	53	28.8	**0.002**	118	39.7	11	9.5	**<0.001**
	Rather yes	38	19.8	21	11.4		57	19.2	5	4.3	
	Undecided	19	9.9	23	12.5		33	11.1	9	7.8	
	Rather no	21	10.9	18	9.8		32	10.8	9	7.8	
	No	24	12.5	51	27.7		32	10.8	55	47.4	
	Missing values	24	12.5	18	9.8		25	8.4	27	23.3	
**Under which** **conditions would you agree to transfer your health data online?** **(Multiple answers** **possible.)**	Data safety	134	69.8	106	57.6	**<0.001**	224	75.4	34	29.3	**<0.001**
	Answer received °	91	47.4	61	33.2		146	49.2	11	9.5	
	Personal advantage	68	35.4	39	21.2		103	34.7	11	9.5	
	Editing personal data	70	36.5	38	20.7		105	35.4	9	7.8	
	Reduce doctor visits	44	22.9	28	15.2		66	22.2	10	8.6	
	Other	5	2.6	2	1.1		8	2.7	0	0	
	Not answered	37	19.3	46	25		33	11.1	64	55.2	
**Can you imagine replacing some doctor appointments by an online consultation?**	Yes	83	43.2	48	26.1	**<0.001**	135	45.5	7	6.0	**<0.001**
	No	87	45.3	127	69.0		144	48.5	93	80.2	
	Missing values	22	11.5	9	4.9		18	6.1	16	13.8	

Bold values denote statistical significance at the *p* < 0.05 level. ° = e.g., for further proceedings, results, course of disease.

## Data Availability

The datasets used and analyzed during the current study are available from the corresponding authors upon reasonable request. The data are not publicly available because they contain information that could compromise research participant privacy.

## Supplementary Materials

The following supporting information can be downloaded at: https://www.mdpi.com/article/10.3390/ijerph191811773/s1, Supplement S1: Translated questionnaire.

## References

[1] NCCN Clinical Practice Guidelines in Oncology. https://www.nccn.org/professionals/physician_gls/default.aspx#head-and-neck.

[2] Amin M.B., Greene F.L., Edge S.B. (2017). AJCC Cancer Staging Manual.

[3] Brierley J., Gospodarowicz M.K., Wittekind C. (2017). TNM Classification of Malignant Tumours.

[4] Hammermüller C., Hinz A., Dietz A., Wichmann G., Pirlich M., Berger T., Zimmermann K., Neumuth T., Mehnert-Theuerkauf A., Wiegand S. (2021). Depression, anxiety, fatigue, and quality of life in a large sample of patients suffering from head and neck cancer in comparison with the general population. BMC Cancer.

[5] Zebralla V., Wichmann G., Pirlich M., Hammermüller C., Berger T., Zimmermann K., Neumuth T., Mehnert-Theuerkauf A., Dietz A., Hinz A. (2021). Dysphagia, voice problems, and pain in head and neck cancer patients. Eur. Arch. Otorhinolaryngol..

[6] Kisser U., Adderson-Kisser C., Coenen M., Stier-Jarmer M., Becker S., Sabariego C., Harréus U. (2017). The development of an ICF-based clinical guideline and screening tool for the standardized assessment and evaluation of functioning after head and neck cancer treatment. Eur. Arch. Otorhinolaryngol..

[7] Söllner W., DeVries A., Steixner E., Lukas P., Sprinzl G., Rumpold G., Maislinger S. (2001). How successful are oncologists in identifying patient distress, perceived social support, and need for psychosocial counselling?. Br. J. Cancer.

[8] Detmar S.B., Muller M.J., Schornagel J.H., Wever L.D.V., Aaronson N.K. (2002). Health-related quality-of-life assessments and patient-physician communication: A randomized controlled trial. JAMA.

[9] Pakhomov S.V., Jacobsen S.J., Chute C.G., Roger V.L. (2008). Agreement between patient-reported symptoms and their documentation in the medical record. Am. J. Manag. Care.

[10] Hutchings H.A., Alrubaiy L. (2017). Patient-Reported Outcome Measures in Routine Clinical Care: The PROMise of a Better Future?. Dig. Dis. Sci..

[11] Büttner M., Zebralla V., Dietz A., Singer S. (2017). Quality of Life Measurements: Any Value for Clinical Practice?. Curr. Treat. Options Oncol..

[12] Duman-Lubberding S., van Uden-Kraan C.F., Peek N., Cuijpers P., Leemans C.R., Verdonck-de Leeuw I.M. (2015). An eHealth Application in Head and Neck Cancer Survivorship Care: Health Care Professionals’ Perspectives. J. Med. Internet Res..

[13] Brettschneider C., Lühmann D., Raspe H. (2011). Informative value of Patient Reported Outcomes (PRO) in Health Technology Assessment (HTA). GMS Health Technol. Assess..

[14] U.S. Department of Health and Human Services Food and Drug Administration (2009). Patient-Reported Outcome Measures: Use in Medical Product Development to Support Labeling Claims. https://www.fda.gov/regulatory-information/search-fda-guidance-documents/patient-reported-outcome-measures-use-medical-product-development-support-labeling-claims.

[15] Chen J., Ou L., Hollis S.J. (2013). A systematic review of the impact of routine collection of patient reported outcome measures on patients, providers and health organisations in an oncologic setting. BMC Health Serv. Res..

[16] Basch E., Deal A.M., Dueck A.C., Scher H.I., Kris M.G., Hudis C., Schrag D. (2017). Overall Survival Results of a Trial Assessing Patient-Reported Outcomes for Symptom Monitoring During Routine Cancer Treatment. JAMA.

[17] Zebralla V., Pohle N., Singer S., Neumuth T., Dietz A., Stier-Jarmer M., Boehm A. (2016). Introduction of the Screening Tool OncoFunction for Functional Follow-up of Head and Neck Patients. Laryngorhinootologie.

[18] Cohen E.E.W., LaMonte S.J., Erb N.L., Beckman K.L., Sadeghi N., Hutcheson K.A., Stubblefield M.D., Abbott D.M., Fisher P.S., Stein K.D. (2016). American Cancer Society Head and Neck Cancer Survivorship Care Guideline. CA Cancer J. Clin..

[19] Broemer L., Friedrich M., Wichmann G., Müller J., Neumuth T., Dietz A., Mehnert A., Wiegand S., Zebralla V. (2021). Exploratory study of functional and psychological factors associated with employment status in patients with head and neck cancer. Head Neck.

[20] Zebralla V., Müller J., Wald T., Boehm A., Wichmann G., Berger T., Birnbaum K., Heuermann K., Oeltze-Jafra S., Neumuth T. (2020). Obtaining Patient-Reported Outcomes Electronically With "OncoFunction" in Head and Neck Cancer Patients During Aftercare. Front. Oncol..

[21] Zebralla V., Wiegand S., Dietz A., Wichmann G., Neumuth T., Mehnert-Theuerkauf A., Hinz A. (2021). Course of Self-Reported Dysphagia, Voice Impairment and Pain in Head and Neck Cancer Survivors. Biology.

[22] Basch E., Deal A.M., Kris M.G., Scher H.I., Hudis C.A., Sabbatini P., Rogak L., Bennett A.V., Dueck A.C., Atkinson T.M. (2016). Symptom Monitoring With Patient-Reported Outcomes During Routine Cancer Treatment: A Randomized Controlled Trial. J. Clin. Oncol..

[23] Denis F., Basch E., Septans A.-L., Bennouna J., Urban T., Dueck A.C., Letellier C. (2019). Two-Year Survival Comparing Web-Based Symptom Monitoring vs Routine Surveillance Following Treatment for Lung Cancer. JAMA.

[24] Denis F., Lethrosne C., Pourel N., Molinier O., Pointreau Y., Domont J., Bourgeois H., Senellart H., Trémolières P., Lizée T. (2017). Randomized Trial Comparing a Web-Mediated Follow-up With Routine Surveillance in Lung Cancer Patients. J. Natl. Cancer Inst..

[25] Denis F., Viger L., Charron A., Voog E., Dupuis O., Pointreau Y., Letellier C. (2014). Detection of lung cancer relapse using self-reported symptoms transmitted via an internet web-application: Pilot study of the sentinel follow-up. Support. Care Cancer.

[26] Denis F., Viger L., Charron A., Voog E., Letellier C. (2014). Detecting lung cancer relapse using self-evaluation forms weekly filled at home: The sentinel follow-up. Support. Care Cancer.

[27] Lizée T., Basch E., Trémolières P., Voog E., Domont J., Peyraga G., Urban T., Bennouna J., Septans A.-L., Balavoine M. (2019). Cost-Effectiveness of Web-Based Patient-Reported Outcome Surveillance in Patients With Lung Cancer. J. Thorac. Oncol..

[28] Wintner L.M., Giesinger J.M., Zabernigg A., Rumpold G., Sztankay M., Oberguggenberger A.S., Gamper E.M., Holzner B. (2015). Evaluation of electronic patient-reported outcome assessment with cancer patients in the hospital and at home. BMC Med. Inform. Decis. Mak..

[29] Jansen F., van Uden-Kraan C.F., van Zwieten V., Witte B.I., Verdonck-de Leeuw I.M. (2015). Cancer survivors’ perceived need for supportive care and their attitude towards self-management and eHealth. Support. Care Cancer.

[30] van Uden-Kraan C.F., Jansen F., Lissenberg-Witte B.I., Eerenstein S.E.J., Leemans C.R., Verdonck-de Leeuw I.M. (2020). Health-related and cancer-related Internet use by patients treated with total laryngectomy. Support. Care Cancer.

[31] ARD/ZDF-Forschungskommission Ergebnispräsentation|ARD/ZDF-Forschungskommission. https://www.ard-zdf-onlinestudie.de/ardzdf-onlinestudie/ergebnispraesentation/.

[32] Kongsved S.M., Basnov M., Holm-Christensen K., Hjollund N.H. (2007). Response rate and completeness of questionnaires: A randomized study of Internet versus paper-and-pencil versions. J. Med. Internet Res..

