# Supplementary material for: Web-Based Patient-Reported Outcomes for ENT Patients—Evaluation of the Status Quo, Patients’ View, and Future Perspectives
Source: Int J Environ Res Public Health. 2022 Sep 18;19(18):11773. doi: 10.3390/ijerph191811773 (PMC9517261; doi:10.3390/ijerph191811773)
Supplement: Supplementary file 1 [file ijerph-19-11773-s001.zip › ijerph-1843291-supplementary.pdf]

International Journal of
Environmental Research and Public Health

MDPI

Supplement S1

Translated questionnaire
(original language: German)

1) What is your reason for consultation?
   a. Tumor Aftercare
   b. Common Consultation Hour. Reason for consultation: ……………………………
2) Age and gender
3) What is your highest degree of education?
   a. Professional training
   b. Technical college
   c. University of applied sciences
   d. University degree
   e. Other
   f. None
4) Do you own a web-enabled device?
   a. Yes
   b. No
5) Do you use the internet?
   a. Yes
   b. No
6) Which operating system do you use?
   a. iOS
   b. Android
   c. Other: ……………………………
   d. I don’t know
7) Can you imagine communicating via APP with your physician/ hospital?
   a. Yes
   b. No
8) How often would you like to answer a questionnaire as a patient with cancer or any other chronic disease?
   a. Once a week or more frequently
   b. At least twice a month
   c. Once a month
   d. Once every two months
   e. Once every three months
   f. Less frequently

9) How much time would you spend on a recurrent online questionnaire?
    a. < 2 min
    b. < 5 min
    c. < 10 min
    d. < 15 min
    e. < 20 min
10) How many questions would you agree to answer regularly?
    a. < 10
    b. 11-20
    c. 21-30
    d. 31-40
    e. 41-50
    f. > 50
11) Do you think you could collect health data (e.g. state of health, heart rate, blood pressure, etc.), if necessary supported by the APP?
    a. Yes
    b. Rather yes
    c. Undecided
    d. Rather no
    e. No
12) Do you think that using an APP in addition to regular doctor's appointments would improve patient care?
    a. Yes
    b. Rather yes
    c. Undecided
    d. Rather no
    e. No
13) Would you use an APP to transfer your health data to your hospital?
    a. Yes
    b. Rather yes
    c. Undecided
    d. Rather no
    e. No
14) Under which conditions would you agree to transfer your health data online? (multiple answers possible)
    a. Data safety
    b. Receiving answer to your data (e.g. further proceedings, results, course of disease)
    c. Personal advantage (e.g. fast appointment)
    d. Possibility of deletion of or access to personal data
    e. Reduction of doctor's appointments
    f. Other: ……………………………

15) Can you imagine replacing some doctor's appointments by an online consultation?
    a. Yes
    b. No
16) Main reasons for being online? (multiple answers possible)
    a. Email
    b. Chatting
    c. Online communities (e.g. facebook)
    d. Research of information
    e. Shopping
    f. Work tasks
    g. Other: ……………………………
17) Do you use the internet for research concerning health?
    a. Yes
    b. No
18) How many hours a day do you spend on the internet for private use on average?
    a. < 30 min
    b. < 1 hour
    c. 1-2 hours
    d. 2-3 hours
    e. 3-4 hours
    f. 4-5 hours
    g. 5-6 hours
    h. > 6 hours
    i. None
19) How many hours a day do you spend on the internet for work-related tasks on average?
    a. < 30 min
    b. < 1 hour
    c. 1-2 hours
    d. 2-3 hours
    e. 3-4 hours
    f. 4-5 hours
    g. 5-6 hours
    h. > 6 hours
    i. None
20) How do you get information concerning your health issue? (multiple answers possible)
    a. Doctors
    b. Friends
    c. Newspaper, magazines
    d. Internet

   e. TV
   f. Other: ……………………………
21) How much time do you spent on the internet for research concerning health issues?
   a. < 1 hour
   b. 1-4 hours
   c. 4-8 hours
   d. 8-16 hours
   e. 16-24 hours
   f. 1-3 days
   g. 3-7 days
   h. > 7 days
22) Please rate the information published on the internet.
   a. 1 (very good)
   b. 2
   c. 3
   d. 4
   e. 5
   f. 6 (deficient)
23) Have you ever visited a medical website?
   a. Yes
   b. No
24) If yes, what medical website did you visit? (multiple answers possible)
   a. Netdoktor.de
   b. Krebsinformationsdienst.de
   c. Patienten-information.de
   d. Washabich.de
   e. Onmeda.de
   f. Other: ……………………………
25) Have you ever visited the website of the Clinic for Otorhinolaryngology, Head and Neck Surgery at the university hospital of Leipzig?
   a. Yes
   b. No
26) If yes, please rate the website.
   a. 1 (very good)
   b. 2
   c. 3
   d. 4
   e. 5
   f. 6 (deficient)

27) Did you search for information on your health issue on the website?
    a. Yes
    b. No
28) Why did you choose our clinic for treatment?
    a. Located nearby
    b. On recommendation of my family doctor
    c. On recommendation of my ENT doctor
    d. On recommendation of other patients
    e. Research on the internet
    f. Other: ……………………………
29) Have you read recommendations on physicians on the internet?
    a. Yes
    b. No
30) What websites have you visited for recommendations on physicians? (multiple answers possible)
    a. Jameda
    b. Sanego
    c. Other: ……………………………
31) Did you search the web for information about your medical treatment/operation?
    a. Yes
    b. No
32) Was the information on the internet helpful for you?
    a. Yes
    b. No
33) Would you like to make appointments via email?
    a. Yes
    b. No
34) Did the information on the internet change your point of view on your health issue?
    a. Yes
    b. No
35) Did you get enough information about your health issue on the internet?
    a. Yes
    b. No
